# How consistent is the determination of the anaerobic threshold among members of the same cardiac rehabilitation team?

**DOI:** 10.3389/fphys.2026.1914264

**Published:** 2026-09-30

**Authors:** Nanaho Minagawa, Kazuo Kondo, Hirotaka Nishijima, Yusuke Mori, Takeo Ikota

**Affiliations:** 1 Shinfuwakai Asabu Heart and Cardiac Rehabilitation Clinic, Sapporo, Japan; 2 Department of Rehabilitation, Japan Healthcare University, Sapporo, Japan; 3 Cardiology, Aishin Memorial Hospital, Sapporo, Japan; 4 CARECOM Co., Ltd., Sapporo, Japan; 5 Cardiac Rehabilitation Center, Caress-Sapporo Caress Memorial Hospital, Sapporo, Japan

**Keywords:** anaerobic threshold, cardiopulmonary exercise test, intraclass correlation coefficient, limits of agreement, observer

## Abstract

**Introduction:**

Consistency in the anaerobic threshold (AT) determination is essential for cardiac rehabilitation teams comprising multiple professionals who conduct cardiopulmonary exercise tests (CPETs) and interpret their results. The effect of years of experience on interpreting CPET results, including the AT, has been examined. However, consistency in the AT determination among members of the same institution with experience beyond the training period has not been reported.

**Methods:**

We evaluated consistency in the AT determination among four members at a cardiovascular hospital (three full-time physical therapists and one part-time consultant physician) with 4 to 30 years of experience with CPETs. Another full-time physical therapist acted as the controller and did not participate in the AT reading. This retrospective study used 260 blinded and randomized CPET data files from 130 patients (130 data pairs comprising inpatient and outpatient data). All observers determined the AT twice using the modified V-slope method.

**Results:**

The mean AT values showed significant interobserver differences (p<0.001) driven by a slightly but significantly lower value determined by the consultant physician. No significant differences in the values determined by the three full-time staff members were observed. Despite interobserver differences during inpatient and outpatient phases, the longitudinal change in the AT did not differ significantly (p=0.58). The intrarater reliability of four observers was good [intraclass correlation coefficient (1,1) = 0.87–0.94], with limits of agreement of 103.6 to 185.0 mL/min. The inter-rater reliability was also good [intraclass coefficient (2,1) = 0.87], with limits of agreement of observer pairs of 139.2 to 220.5 mL/min. Reliability was not associated with years of experience.

**Conclusions:**

Good consistency in the AT determination is achievable in a clinical setting. Adherence to a single standardized method across the team likely facilitates this consistency and provides a reliable objective measure for monitoring exercise tolerance.

## 1 Introduction

The terms anaerobic threshold (AT), gas exchange threshold, ventilatory threshold, and lactate threshold are used in the context of exercise intensity during an incremental exercise test when lactate levels in the blood begin to increase compared with those at rest. Lactic acid (lactate + H^+^) is buffered by the bicarbonate ion (HCO_3_^−^) in the blood to form carbon dioxide (CO_2_). This excess CO_2_ is distinct from the CO_2_ generated by mitochondrial energy metabolism. The point in the exercise test when this excess CO_2_ is generated is termed the AT (Wasserman et al., 2012).

The AT has been used for decades as an index of exercise tolerance, and more recently its clinical application has expanded to pre-operative risk assessment (Older et al., 1993, 1999; Lockhart et al., 2022; Wijma et al., 2025; Beg et al., 2026), where substantial evidence supports the use of AT for predicting postoperative morbidity and mortality. However, the current gold standard for assessing the effects of exercise and predicting cardiac disease prognosis is peak oxygen uptake (VO_2_) during symptom-limited maximal exercise (Balady et al., 2010), as it is supported by substantially more evidence for these purposes and exhibits less variability and less uncertainty of measurement compared with AT. Yet, peak VO_2_ requires the maximal effort by the patient. On the other hand, AT does not require maximal effort by the patient because it is detected well before maximal effort. AT may serve as a useful submaximal marker of aerobic exercise capacity together with oxygen uptake efficiency slope (OUES) (Baba et al., 1996). However, because the detection and determination of AT are subjective and dependent on the observer, highly reliable measurement is required. Furthermore, the patient’s identifiers, clinical information, type of intervention, and pre- versus post- intervention status must be fully blinded to the assessor before the AT determination can be considered trustworthy (Day and Altman, 2000; Grocott and Pearse, 2010). The most prevalent method of detecting the AT is considered the V-slope method (Wasserman et al., 2012), which uses the VCO_2_ vs. VO_2_ graph. Two versions of the V-slope method exist, one reported by Beaver et al. (1986) and one reported by Sue et al. (1988). However, some use alternative methods based on ventilatory equivalents and end-tidal gas values to detect the AT (Caiozzo et al., 1982). The relevant graphs must be subjectively assessed by the observers to determine the AT. Commonly, one or two administrators perform the cardiopulmonary exercise test (CPET) and explain the results to each patient at the site. Therefore, consistency within the institution is critical. This practical aspect of AT determination has not been previously assessed. Therefore, this study aimed to evaluate consistency in the determination of the AT among four observers (CPET administrators/supervisors) at one institution and examine the extent to which differences in the reproducibility of the AT assessment by individual observers may affect the observed longitudinal changes in the AT over time (such as the training effect). This study was not conducted at a university hospital or other research institution; instead, it included on-site health professionals (physical therapists and a physician) involved in cardiac rehabilitation at a general cardiovascular hospital, thus distinguishing it from previous studies on AT determination. Potentially important factors influencing the reproducibility of AT determination, such as scientific training and the learning environment, may differ between academic centers and general hospitals.

## 2 Methods

### 2.1 Observer

All four full-time physical therapists and one part-time consultant physician at Hokko Memorial Hospital in Sapporo, Hokkaido, Japan, participated in this study. One physical therapist participated as the controller and was assigned to randomize and blind the data to ensure complete blinding. The controller did not determine the AT. The observers who assessed the AT were three physical therapists and a consultant physician who were involved in cardiac rehabilitation during their daily practice, conducted CPETs, and explained the results to patients. The mean number of CPETs performed during the data collection period was 163.5 per year (approximately 40 tests per year per person while the controller was on regular duty). The three observers had been performing CPETs for 17 years, 7 years, and 4 years. The consultant physician had approximately 30 years of experience conducting CPETs and assessing the AT at various institutions. Therefore, it was not possible to accurately quantify his cumulative experience in assessing AT.

### 2.2 CPET database

Of the 994 consecutively admitted patients with cardiovascular disease who underwent CPETs at Hokko Memorial Hospital between 2010 and 2020, the clinical details of 130 patients with available data regarding both the inpatient and maintenance outpatient phases (n = 260 data files) were extracted retrospectively. The number of patients required was calculated based on a previous study conducted at Hokko Memorial Hospital (Nishijima et al., 2019). As the change in the AT between the inpatient and maintenance outpatient phases was approximately 60 mL/min (± 200 mL/min), we aimed to use this difference in our calculations and set the significance level at 5% and power at 80%. The required sample size was determined to be 130, after considering the presence of undetectable ATs. The interval between the inpatient and maintenance outpatient phases was 3 to 6 months for all patients.

### 2.3 Exercise testing and cardiac rehabilitation

Exercise tests were performed using an upright bicycle with a breath-by-breath gas analyzer (AE300S; Minato Ikagaku, Tokyo, Japan). The calibration for the flowmeter and gas analyzer was performed before each CPET. The CPET protocol included a ramp rate of 10 W/min for 94% of cases; however, ramp rates of 5 W/min and 20 W/min were also used for some cases. The work rate was determined based on the clinical state of the patient so the ramp exercise would end approximately at 10-minute. The exercise test was submaximal. Once AT has been identified, the exercise could be terminated by the test supervisor depending on the patient’s clinical condition and appearance. The criteria for early termination of included the appearance of ventricular premature beats, systolic blood pressure of 200mmHg, inability of the patient to maintain predetermined pedaling rate. The protocols used during the inpatient and maintenance outpatient phases were identical. The cardiac rehabilitation was conducted according to the Guideline of the Japanese Association of Cardiac Rehabilitation (Japanese Association of Cardiac Rehabilitation, 2021). The exercise prescriptions were individualized and based on the measured AT.

### 2.4 Implementation of the blinded AT assessment

First, breath-by-breath original patient data (one CSV file per patient) were transferred to Microsoft Excel (Redmond, WA, USA) and converted to moving means of eight breaths. A V-slope graph was created using the VO_2_ and VCO_2_ values. Then, the 260 patient data files were randomized using a computer program that replaced the file names with randomized numbers. Additionally, personal information included in the file, such as identification numbers, names, height, weight, and measurement dates and times, were deleted. Two types of blinded files were needed to assess intraobserver reliability; therefore, randomization was performed twice. Two folders, one for the first reading and another for the second reading, were prepared for the analysis. Randomization of the whole set of 260 data files (130 inpatient phase files and 130 maintenance outpatient phase files) was performed. Deleting personal identifiers and creating a V-slope graph in each file were achieved by using a custom macro written in Visual Basic for Applications (VBA) within Microsoft Excel.

Next, the observers assessed the AT using the V-slope method for the first analysis folder. Using the V-slope method, a line parallel to the diagonal in the graph of VCO_2_ vs. VO_2_ (at a 45° angle to the x-axis) was drawn through the data points and used as the baseline, and the breakpoint deviating from this baseline was judged as the AT (**Figure 1**). This modified V-slope method was devised by Sue et al. (1988) and used for all case presentations in the textbook by Wasserman et al. (2012) as well as in the multicenter study by Myers et al. (2010), which is the largest AT reliability study. Although this method has been used at our facility for AT determination in daily practice, we reconfirmed it before distributing the data to the four observers for the AT assessment. The use of this method as a primary method of determining AT was decided at our internal department meeting 3 years before this current study. The AT values determined by the observers were entered into a separate Excel file (Microsoft Excel 2024) and collected by the controller when all first analyses had been completed. The same AT determination method was used for the second folder of blinded case files. The AT determination period was set at 3 months for the first and second AT determinations. Finally, the controller de-randomized and unblinded the data collected from each observer and performed the analysis.

**Figure 1:**
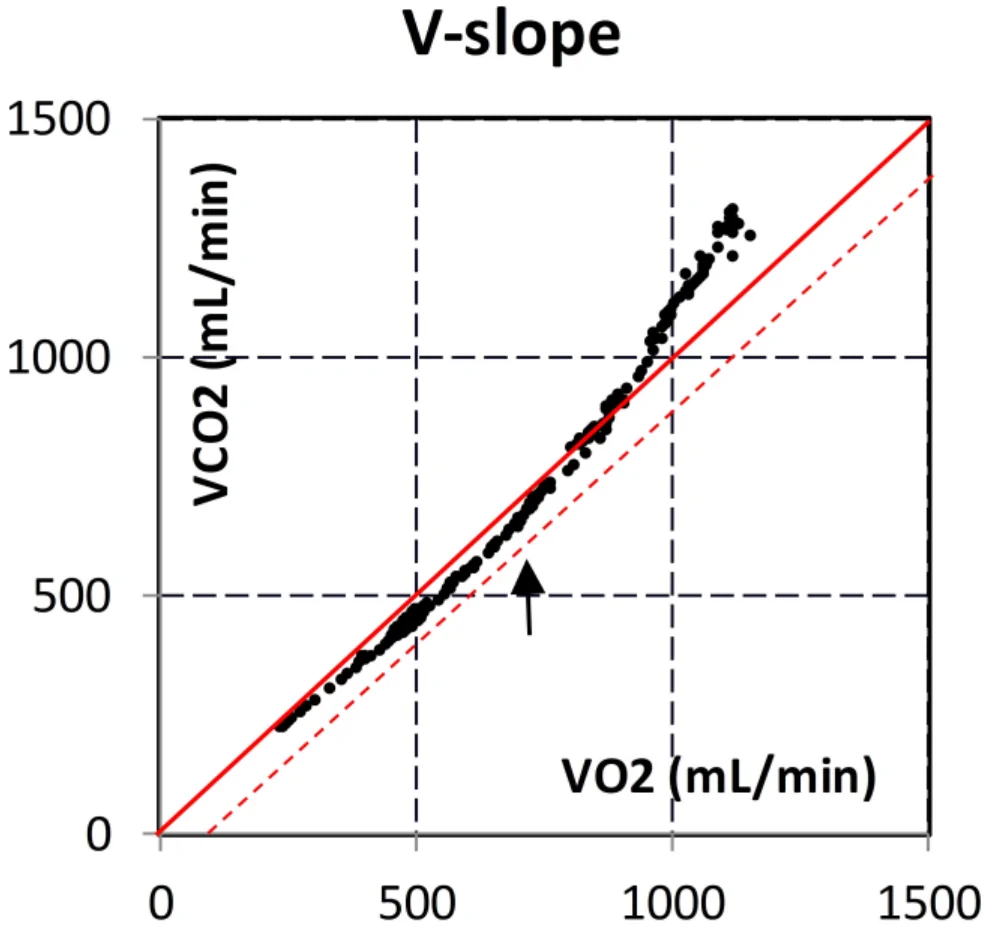
Representative example plots of V-slope graph. We used the modified V-slope method devised by Sue et al. (1988), in which a line parallel to the diagonal (at a 45° angle to the x-axis) is drawn through the data points on the VCO_2_ vs. VO_2_ graph to serve as a baseline. Breakpoints deviating from this baseline were judged as AT.

### 2.5 Statistical analysis

Data obtained from the CPET were analyzed to determine the work rate, Borg rating of perceived exertion scale score, blood pressure, heart rate, VO_2_, VCO_2_, and R at the end of the study. As the data collected in the study were examined retrospectively and not all exercise tests were performed up to symptomatic limitation, the values at the end of the exercise were adopted (denoted as the end-exercise value). During the AT assessment, the actual values determined by each observer as well as the number of cases for which a determination was possible (determination rate) and rate of indeterminate cases were noted. The difference in the AT between the inpatient and maintenance outpatient phases was also calculated.

The AT determinations by the four observers were compared using a repeated measures analysis of variance (ANOVA). Interobserver reliability, intraobserver reliability, and the margin of error of the AT were also assessed. The effects of these parameters on changes in the AT over time were also examined. Both intraclass correlation coefficients (ICCs) [i.e., ICC(2,1) and ICC(1,1)] were calculated for interobserver reliability, and Bland–Altman limits of agreement (LOA) were also calculated (Bland and Altman, 1986). The coefficient of variation for the error was calculated using the within-subject standard deviation method (Myers et al., 2010). Statistical analyses were performed using R software version 4.1.2 (R Foundation for Statistical Computing, Vienna, Austria). The significance level was set at 5% (p < 0.05). The ICC evaluation criteria followed those reported by Koo and Li (2016).

### 2.6 Ethics approval and consent to participate

Approval to conduct this study was obtained from the Institutional Review Board of Hokko Memorial Hospital (ethical approval no. K-45). This study was conducted in accordance with the Declaration of Helsinki and Japanese Ethical Guidelines for Medical and Health Research involving Human Subjects. The data handled in this study were anonymized by the controller, who removed personal information and assigned random numbers so that individuals could not be identified. A table of correspondence between individuals and random numbers was maintained by the controller. CPET was routinely performed after verbally explaining the purpose and methods to the participants, and informed consent was obtained from all participants. All observers were informed of the research methods at the beginning of the study, understood the purpose of the study, and agreed to participate as observers. Additionally, the observers did not discuss their AT determinations with each other.

## 3 Results

### 3.1 Patient baseline characteristics and AT determination rate

The baseline characteristics of the 130 patients in our cohort are shown in **Table 1**. Most of the patients had ischemic heart disease or heart failure and had undergone open heart surgery. The mean duration of the CPET from the inpatient phase to the maintenance outpatient phase was 126.4 days (± 25.7 days). The basic parameters of the CPET are shown in **Table 1**. The AT determination rates ranged from 90.4% to 96.2% for each of the four observers for the 260 case files, and all observers were able to determine the AT for 225 case files (86.5% of all cases) (**Table 2**). Across inpatient and maintenance outpatient phases (130 cases), the AT determination rate ranged from 83.1% to 92.3% for each of the four observers for the 130 patients, and all observers were able to determine the AT for 99 of the 130 patients (76.2% of all patients). The reason for the failure to determine AT was available from comments left by the physician assessor (the total 260 cases, the first reading): dysfunctional breathing pattern, 3; Failure to reach AT, 5; technical data quality issues, 3.

**Table 1:** Characteristics of the study population and final values of the cardiopulmonary exercise test.

Characteristic	Total cohort (n = 130)	
Age (years)	65 ± 11	
Sex (male/female)	111/19	
Body height (cm)	165.6 ± 11.3	
Body weight (kg)	65.4 ± 11.4	
BMI	23.9 ± 3.1	
Days between inpatient and maintenance outpatient phases (day)	126.4 ± 25.7	
Underlying condition (%)		
Postoperative period of open heart surgery	31	
Coronary artery disease	42	
Heart failure	25	
Others	2	
Medications (%)		
β-blockers	52	
ACE inhibitors	23	
ARB	29	
Ca antagonists	33	
Diuretics	49	
Inotropics	8	
Antidiabetic drugs	28	
Lipid-lowering drugs	58	
End-exercise values of cardiopulmonary exercise test	Inpatient phase	Maintenance outpatient phase
Work rate (watt)	66.5 ± 20.2	82.8 ± 26.9
Borg RPE scale: shortness of breath	13.1 ± 2.0	13.3 ± 2.0
Borg RPE scale: leg fatigue	14.0 ± 2.1	14.8 ± 2.0
Heart rate (bpm)	110.8 ± 23.3	125.8 ± 110.7
Oxygen uptake (mL/min)	858.6 ± 262.3	1044.7 ± 361.6
Oxygen uptake based on weight (mL/min/kg)	12.8 ± 3.2	15.6 ± 3.8
Respiratory exchange rate	1.14 ± 0.10	1.18 ± 0.12

Data are presented as the mean ± standard deviation or number of patients (%).

ACE, angiotensin-converting enzyme; ARB, angiotensin receptor blocker; BMI, body mass index; Ca, calcium; RPE, rating of perceived exertion (Borg RPE scale of 20).

**Table 2:** Determination of the anaerobic threshold.

Observer	Combined data (n=260)			Data pairs (n=130)		
	Cases deter-mined	Rate (%) determined	Rate (%) undeter-mined	Cases deter-mined	Rate (%) determined	Rate (%) Undeter-mined
Observer a	249	95.8	4.2	120	92.3	7.7
Observer b	238	91.5	8.5	110	84.6	15.4
Observer c	235	90.4	9.6	108	83.1	16.9
Observer d	250	96.2	3.8	120	92.3	7.7
All observers*	225	86.5	13.5	99	76.2	23.8

*All observers determined the anaerobic threshold.

### 3.2 AT determination consistency, inter-observer and intra-observer reliability

A significant difference in the mean AT values (mL/min) determined by the four observers was observed (inpatient phase: range, 562–584; ANOVA p=0.001; maintenance outpatient phase: range, 631–666; ANOVA p<0.001). The multiple comparisons test revealed that the difference was attributable to the value determined by the consultant physician; however, this difference was small (range, 20–30 mL/min). No significant difference among the values determined by the three physical therapists was observed. Additionally, the differences over time were not significant (range 55–74; p=0.58) (**Table 3**).

**Table 3:** Relationship between intraobserver reliability and change over time between the inpatient and maintenance outpatient phases.

Observer	ICC (1,1) (95%CI)	LOA (mL/min)	CV %	Mean AT of two determinations (mL/min)			Difference: mean AT between phases (mL/min)	p
				All data	Inpatient	Outpatient		
Obs a	0.94 (0.93-0.96)	126.0	7	599.0 ± 185.9	562.8 ± 170.0	631.1 ± 194.2	66.6	*
Obs b	0.93 (0.91-0.95)	103.6	6	619.7 ± 193.7	579.1 ± 171.2	655.7 ± 205.7	55.4	*
Obs c	0.87 (0.83-0.89)	185.0	11	627.3 ± 172.9	583.1 ± 154.5	666.5 ± 179.4	66.2	*
Obs d	0.94 (0.92-0.95)	135.0	8	626.7 ± 186.3	584.9 ± 172.7	663.7 ± 190.7	74.3	*
ANOVA multiple compar-ison				p<0.001 Obs a vs. b, c, and d	p=0.001 Obs a vs. b, c, and d	p<0.001 Obs a vs. b, c, and d	p=0.583	

*p<0.001, inpatient vs outpatient.

ANOVA, analysis of variance; CV, coefficient of variation; ICC, intraclass correlation coefficient; LOA, limits of agreement; Obs, observer.

Intraobserver reliability, ICC (1,1) ranged from 0.87 to 0.94 (95% confidence interval, 0.83–0.96) for the four observers, and LOA ranged from 103.6 to 185.0 mL/min (**Table 3**, **Figure 2**). Interobserver reliability was investigated in 99 cases (198 case files); a determination was possible for all observers, with ICC (2,1) of 0.87 (95% confidence interval, 0.84–0.90) (**Table 4**, **Figure 3**). The mean change in the AT between the inpatient and maintenance outpatient phases (AT in the maintenance outpatient phase minus AT in the inpatient [mL/min]) was 66.6 mL/min (± 121.4 mL/min; n=99 patients) for all four observers, and the individual data for each observer also showed significant differences over time (p<0.001). The change in the AT over time showed no significant differences between observers (p=0.58) (**Table 3**). These results were not related to the observers’ years of experience, ICC, or LOA.

**Figure 2:**
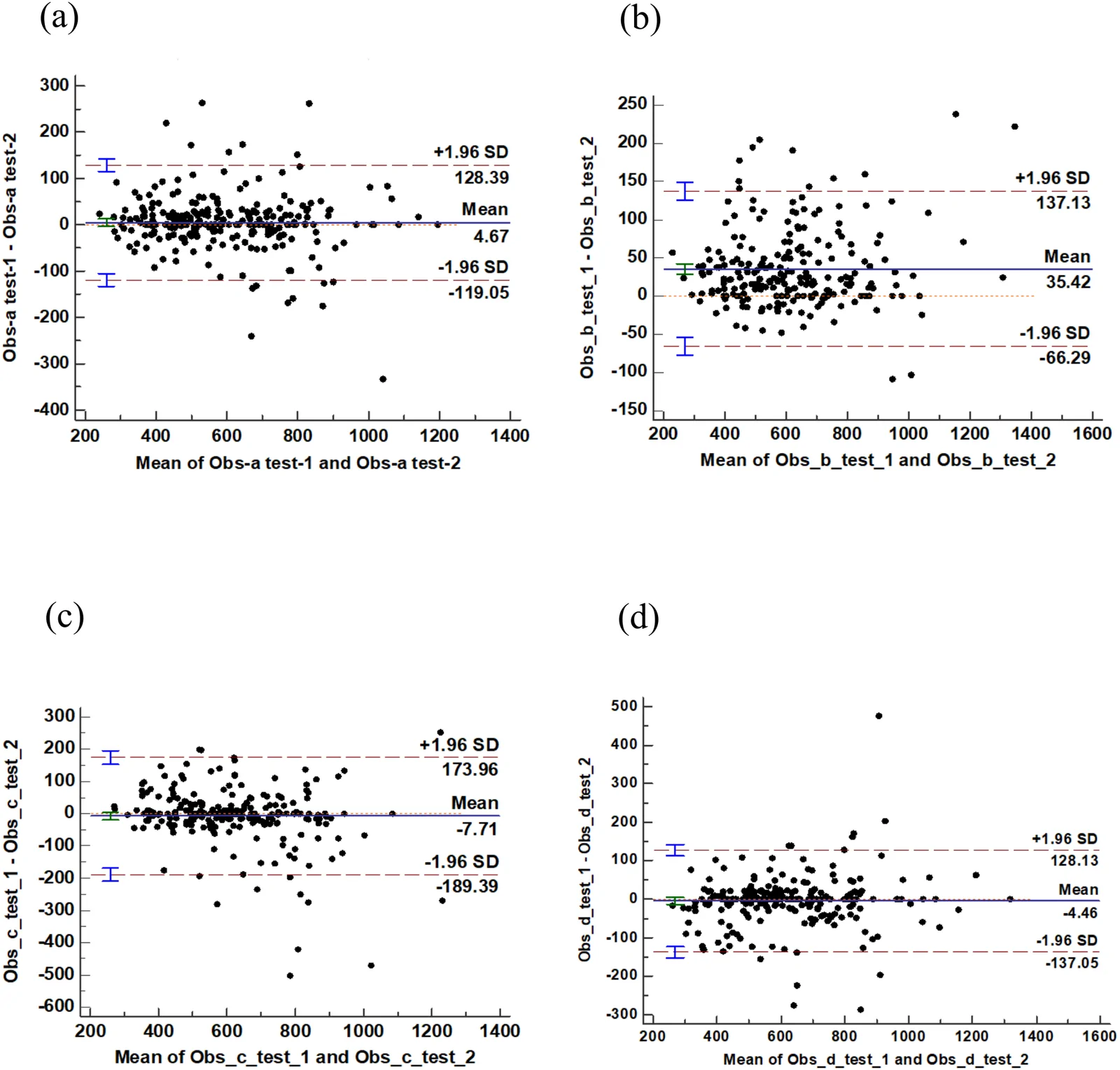
A Bland-Altman plot comparing the results of two measurements of AT assessed by four observers: **(a)** Observer a, **(b)** Observer b, **(c)** Observer c, and **(d)** Observer d. The x-axis shows the mean values of the two measurements, and the y-axis shows the difference between the measurements. The solid line represents the mean and the broken line the ±1.96 SD. The limits of agreement (LOA) shown in **Table 3** were calculated using the within-subject standard deviation (Sw) method (1.96 * sqrt(2) * Sw) according to Myers et al. (2010), whereas this figure plots the direct standard deviation of the differences (mean difference ± 1.96 SD), accounting for the minor differences in values.

**Table 4:** Interobserver reliability.

Interobserver reliability	n	ICC (2,1) (95% CI)	Mean AT (mL/min)	Difference (mL/min)	LOA (mL/min)	CV (%)
Obs a vs. Obs b	236	0.92 (0.84-0.95)	619.2	35.5	139.4	8
Obs a vs. Obs c	233	0.83 (0.78-0.87)	608.8	18.4	203.5	12
Obs a vs. Obs d	245	0.89 (0.86-0.92)	607.4	22.3	166.0	10
Obs b vs. Obs c	226	0.86 (0.82-0.89)	631.1	14.9	192.1	11
Obs b vs. Obs d	236	0.93 (0.91-0.95)	629.0	12.7	139.2	8
Obs c vs. Obs d	234	0.81 (0.76-0.85)	620.6	2.0	220.5	13
All observers	198	0.87 (0.84-0.90)				

“All observers” refers to the ICC calculated using the measurements from all four assessors (A, B, C, and D) simultaneously, rather than to an average of the six pairwise ICCs. The six pairwise ICCs represent the interobserver reliability for each of the six possible assessor pairs, whereas the “all observers” ICC represents the overall interobserver reliability across all four assessors.

AT, anaerobic threshold; CI, confidence interval; CV, coefficient of variation; ICC, intraclass correlation coefficient; LOA, limits of agreement; Obs, observer.

**Figure 3:**
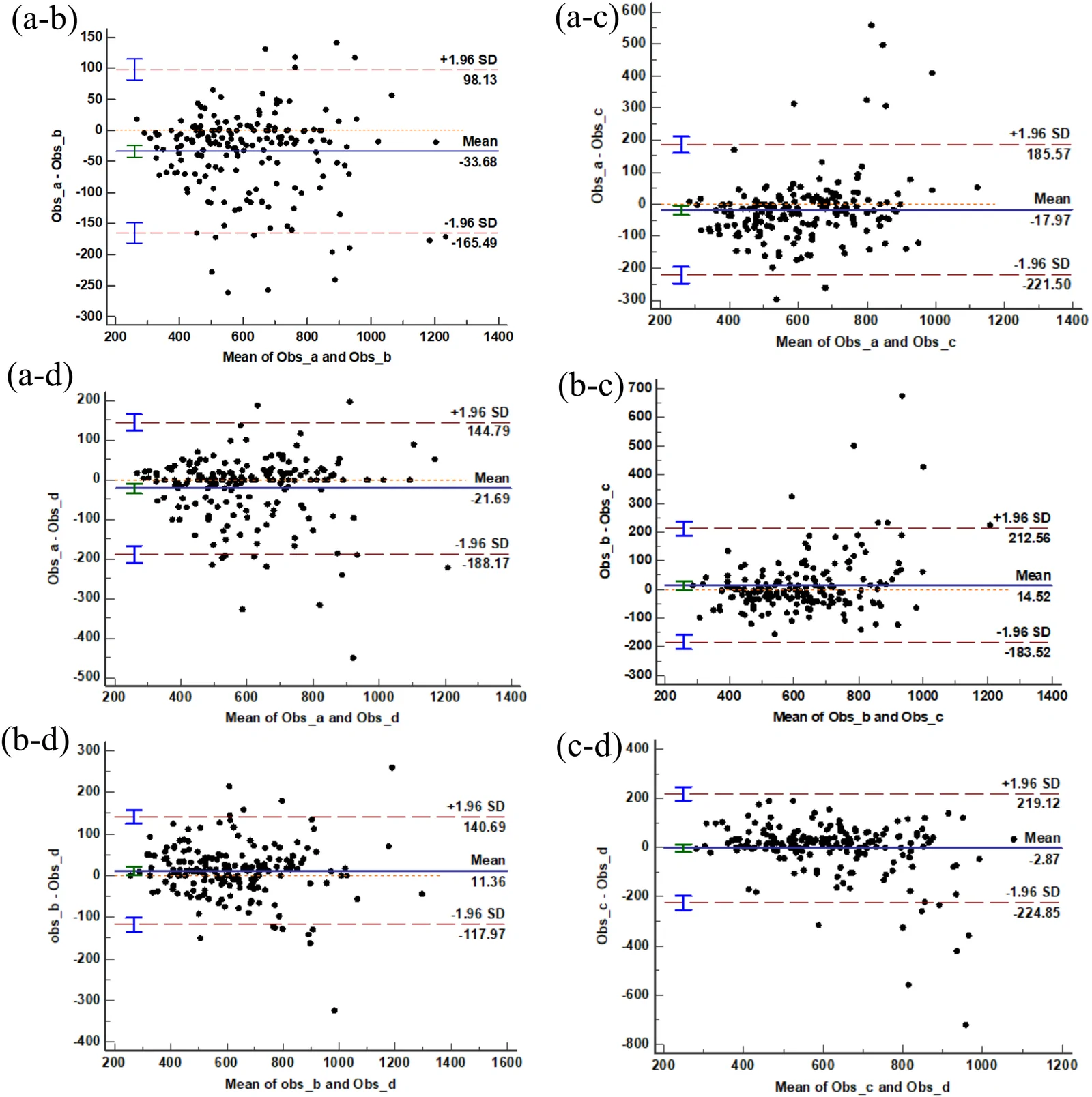
Inter-rater Bland-Altman plot comparing AT measurement results in pairs of two observers: **(a, b)** Obs a vs. Obs b, **(a–c)** Obs a vs. Obs c, **(a–d)** Obs a vs. Obs d, **(b, c)** Obs b vs. Obs c, **(b–d)** Obs b vs. Obs d, and **(c, d)** Obs c vs. Obs d. The x-axis shows the mean of the measurements for each pair, and the y-axis shows the difference between the measurements. The solid line represents the mean, and the broken line ±1.96 SD. The Bland-Altman plots in **Figure 3** are based on the listwise-complete dataset (n=198 cases where all four observers successfully determined the AT) to maintain a consistent sample across all subplots, which accounts for the minor differences in mean differences and LOAs compared to the pairwise-complete results reported in **Table 4**.

## 4 Discussion

### 4.1 Consistency in the AT determination

Not all the parameter outputs from the CPET are automatic. Among them, the AT determination is probably the most difficult measurement that CPET supervisors must establish, and very often they make this determination alone. No standard or universally accepted method of automatic AT detection is currently available. Obtaining assistance from an experienced assessor or peer group discussion is often not possible at the site. In actual clinical practice, the data must be interpreted and explained to the patient at the site. In this setting, consistency in the AT determination among members of the team is critical. This factor was the motivation for this study. Intraobserver reliability across the four observers was good. However, a significant difference in the mean value of the AT among the four observers was observed. This difference was mainly attributed to the difference between the value determined by the part-time consultant physician and the values determined by the three full-time physical therapists. The three full-time staff members worked together every day. Although all four observers used the same modified AT detection method, the consultant physician lacked the everyday experience of working with the three physical therapists. This may have resulted in a statistically significant difference in AT readings, despite the small magnitude of the difference (20–30 mL/min) from a clinical and biological perspective. One possible explanation is the inherently subjective nature of identifying the precise breakpoint of AT. The transition from a presumed pre-AT baseline to an adopted AT data point may be gradual rather than occurring at a single, clearly identifiable data point. Consequently, one assessor may tend to identify an earlier point in the transition, whereas another may select a later point. Although standardized training and procedures may reduce such differences, some degree of subjectivity in AT determination may be difficult to eliminate completely. As expected, the change between the inpatient phase and outpatient phase was not affected, and no significant difference was observed among the participants. Good team consistency in the AT determination likely resulted from the method of AT determination (V-slope method of Sue et al. (1988)) and agreement among the team members that this study was necessary. The modified V-slope method is relatively simple to perform because a breakpoint is often apparent on the monitor during testing. To our knowledge, no similar study has been reported in the literature in which staff members from the same cardiac rehabilitation unit assessed the consistency of AT determination within their own team in a real-world clinical setting. In contrast, it appears that previously reported studies have generally involved different investigators specifically recruited to assess the reproducibility of AT determination in general.

Reproducibility of observers with experience

One of the factors associated with the AT determination was experience. Dolezal et al. (2017) compared the AT assessments of two experienced instructors and four novice respiratory fellows who knew the basic concepts but had no experience with determining the actual AT. The variability of their judgments (95% LOA: two standard deviations of the difference between the two determinations [mL/min]) (Bland and Altman, 1986) widened from approximately 195 mL/min for the two expert assessors to approximately four-times more when the AT assessments determined by the four novice assessors were included. Kaczmarek et al. (2019) compared the AT assessments of two experienced physicians with those of several qualified medical assistants. The 95% LOA spread from 249 mL/min among physicians to just less than twice that when the assessments of the assistants were included. Variability in the AT assessment by our assessors with at least 4 years of experience with AT determination was similar.

### 4.2 Reported reproducibility of the AT determination

Our review of the literature included the following inclusion conditions: there was the statement that the AT determination was blinded and LOA was considered. This was for the comparison with our report. Dolezal et al. (2017) performed three CPETs for 10 healthy participants, and the analysis showed 95% LOA of 195 mL/min (without the ICC calculation). Kaczmarek et al. (2019) performed CPETs for 1079 healthy participants and reported 95% LOA of 249 mL/min and ICC of 0.901. In a multicenter study, Myers et al. (2010) performed four cycles of CPETs for 428 patients with heart failure and reported intraobserver reliability (95% LOA) of 164 mL/min to 234 mL/min and interobserver reproducibility (95% LOA) of 207 mL/min to 247 mL/min. Shimizu et al. (1991) reported an ICC of 0.93 for 17 patients with cardiovascular disease and 6 healthy participants. Dubé et al. (2016) reported that the V-slope method resulted in an ICC of 0.98 for healthy participants (n=23) and ICC of 0.92 for patients with chronic obstructive pulmonary disease (COPD) (n=92) as well as a lower ICC of 0.35 to 0.78 for patients with more advanced COPD. Reproducibility appeared to be reduced among patients with advanced COPD. This was probably because patients with disease tend to exhibit greater respiratory irregularity or noise, which may make the plots less suitable for detecting AT and thus reduce the reproducibility of AT determination. An accepted or standard reference value is not available for the ICC or LOA. However, our ICC and LOA values were within the range reported in the literature. Lastly, the reliability of AT and other CPET parameters for preoperative risk assessment, a relatively recent application of CPET, has received increasing attention and has been extensively investigated (Rose et al., 2022; Silvapulle and Darvall, 2022). CPET is increasingly used in the preoperative evaluation of patients undergoing major surgery because it provides an objective assessment of functional and cardiopulmonary capacity and may help identify patients at increased risk of postoperative morbidity and mortality. Among the various CPET-derived variables, AT has particular clinical relevance because it provides a submaximal measure of aerobic exercise capacity and can be obtained even when a symptom-limited maximal exercise test is not feasible. Thus, the reliability of AT determination is important not only for exercise prescription and evaluation in cardiac rehabilitation, but also for its potential application in clinical risk stratification. Because AT determination is partly dependent on the assessor, variability among assessors may affect the reliability of this parameter and, consequently, its clinical usefulness in settings such as preoperative risk assessment.

### 4.3 Limitations

First, as described in the Methods section, we did not perform symptom-limited maximal exercise testing. In our usual practice, patients were not routinely encouraged to exercise to their symptom-limited maximum. Once AT had been identified, the test could be terminated by the supervising physiotherapist, depending on the patient’s clinical condition and overall appearance. At that point, we had already obtained an important index of aerobic exercise capacity, namely AT. This approach was considered to reduce the physiological and psychological burden on patients and, presumably, to enhance safety, although we acknowledge that a safety advantage of this approach has not been demonstrated by direct clinical evidence. However, it is well established that plasma catecholamine levels increase markedly above AT (Peinado et al., 2014; Shah et al., 2019), potentially increasing the risk of ventricular arrhythmias. The feasibility of a submaximal approach therefore depends on whether AT provides a sufficiently valid index of aerobic exercise capacity in comparison with peak VO_2_. Second, in this study, AT determination was performed only using the modified V-slope method of Sue et al. (1988). Therefore, it was not possible to assess the AT determination when other methods or combinations of methods were used. Generally, the end-tidal partial pressures or concentrations of O2 and CO2 and ventilatory equivalents of VO2 and VCO2 are also used simultaneously with the V-slope method (Balady et al., 2010; Wasserman et al., 2012). Whether such a three-way decision method is more reliable should be examined. Additionally, consultation among observers and averaging of the results of more than one assessor have been conducted in the past report (Bensimhon et al., 2008). Whether this process produces better results has not been proven. Third, Although the patients in our study underwent CPET during the inpatient and maintenance outpatient phases, few of them received continuous outpatient rehabilitation during this period, and the majority received only exercise prescriptions and unmonitored exercise therapy at the time of hospital discharge. Therefore, the results do not reflect the therapeutic effect of a formal exercise program. Lastly, The New York Heart Association functional class was not recorded in the medical database. However, the physical therapist in charge of the cardiac rehabilitation program speculated that most patients in the study cohort had New York Heart Association class I or II.

## 5 Conclusion

This study addressed the issue of consistency in AT readings among our team working in a cardiac rehabilitation unit of a general cardiovascular hospital. Consistency in the AT reading was considered good in terms of Bland–Altman’s LOA and ICC. Awareness and agreement among all members of the rehabilitation unit regarding the need for this type of real-world study enabled this study using only one method of AT detection (modified V-slope method) and contributed to team consistency.

## Data Availability

The datasets presented in this study can be found in online repositories. The names of the repository/repositories and accession number(s) can be found in the article/supplementary material.
